# Distributions of Lanostene-Derived Triterpenoids and Glucan Content in the Fruiting Bodies of the Australian *Ganoderma* Species

**DOI:** 10.3390/jof10100723

**Published:** 2024-10-18

**Authors:** Aline De Oliveira Campos, Mark D. Harrison, David L. Marshall, Peter James Strong

**Affiliations:** 1 Center for Agriculture and the Bioeconomy, Queensland University of Technology, Brisbane 4000, Australia; aline.deoliveiracampos@hdr.qut.edu.au (A.D.O.C.); md.harrison@qut.edu.au (M.D.H.); 2 School of Biology and Environmental Science, Queensland University of Technology, Brisbane 4000, Australia; 3 School of Mechanical, Medical, and Process Engineering, Queensland University of Technology, Brisbane 4000, Australia; 4 Central Analytical Research Facility, Queensland University of Technology, Brisbane 4000, Australia; d20.marshall@qut.edu.au

**Keywords:** ganoderic acids, *Ganoderma* mushrooms, mass spectrometry, metabolite, morphology, triterpenoid

## Abstract

Lanostene-derived triterpenoids and β-glucans are important metabolites in *Ganoderma* mushrooms associated with benefits to human health. The medicinal value of the Australian *Ganoderma* species remains unclear, with no data on triterpenoid distribution or glucan content. In the present study, 22 Australian *Ganoderma* specimens were analyzed for triterpenoid and glucan contents. Thirty-two triterpenoids were identified in the fruiting bodies of 19 of the specimens. Distinct patterns in triterpenoid distribution between laccate and matte fruiting bodies were observed, leading to the classification of four groups of *Ganoderma*. Most of the glucans in the *Ganoderma* fruiting bodies were β-glucans (~99%), with a nominal α-glucan content (~1%). The β-glucan content ranged from 19.5 to 43.5% (*w*/*w*). A range of antioxidant activities was observed for methanol extracts using the ABTS (1.8 to 8.4 mg GAE.g^−1^), DPPH (1.7 to 9.4 mg GAE/g^−1^) and FRAP (24.7 to 111.6 mmol FeSO_4_.g^−1^) assays, with four specimens presenting relatively high radical scavenging and reducing activities. For the first time, we demonstrated that Australian *Ganoderma* mushrooms contain medicinal triterpenoids, including ganoderic acid A, and we established a link between its distribution and the fruiting body morphology. However, further research is required to isolate diploid clones and determine factors that impact triterpenoid and glucan synthesis in these strains.

## 1. Introduction

*Ganoderma* species are saprophytic bracket fungi renowned for containing medicinal compounds [1]. The most studied species, *Ganoderma lucidum*, commonly known as Reishi or Lingzhi, has been used in Eastern medicine for over 2000 years. Reishi mushrooms are cultivated commercially for use in liquid concentrates, powders, and capsules for dietary supplements, cosmetics, and nutraceuticals [1]. *Ganoderma* fruiting bodies typically grow as shelf-like bracket structures with stipitate or sessile (no stipe) forms. The surface of the pileus (cap) can be shiny (laccate) or matte and range from reddish to dark brown, while the base tends to range from creamy to light brown [2]. Traditionally, macroscopic features of the basidiocarps were used to distinguish between two species: *G. lucidum* and *G. applanatum*. However, subsequent research revealed that these traits resulted from parallelism or convergence among genetically distant or non-monophyletic species [3]. The pleomorphic characteristics of mushrooms (e.g., size, color, and shape) offer limited taxonomic information as these are influenced by environmental conditions (e.g., humidity, gas exchange, and light) during fruiting body development [4,5]. Subsequently, the development of the fruiting body impacts metabolite production and its commercial value. To date, the complex nomenclature is used to categorize *Ganoderma* species based on morphological attributes. For example, the term “*G. lucidum* complex” commonly refers to shiny (laccate) dark-reddish fruiting bodies, while “*G. applanatum* complex” is used to describe specimens displaying matte-brown fruiting bodies [6].

Numerous (>900) triterpenes, meroterpenoids, steroids, fatty acids, lectins, phenolics, alkaloids, nucleosides, and polysaccharides have been isolated from *Ganoderma* fruiting bodies, mycelia, and spores [1]. Among these metabolites, lanostene-derived triterpenoids (LDTs) and β-glucans are the most abundant and bioactive components. These compounds, both individually and collectively, have demonstrated anti-tumor, anti-inflammatory, antioxidant, immunomodulatory, anti-diabetic, anti-viral, and anti-bacterial properties, both in vitro and in vivo [7]. Several studies have demonstrated that *Ganoderma* extracts reduce oxidative damage and can ameliorate the impact of reactive oxygen species and promote anti-aging, anti-inflammatory, hepato-protective, neuroprotective [7], nephroprotective [8], and pancreato-protective [9] effects in vivo.

Lanostene-derived triterpenoids are complex molecules consisting of 24 to 32 tetracyclic and pentacyclic carbons, including ganoderic acids (GAs), ganoderenic acids (GNs), lucidenic acids (LAs), applanoxidic acids (AAs), and elfvingic acids (EAs) [10]. Ganoderic acid A (GA-A) is notable among the LDTs found in *G. lucidum* due to its anti-tumor properties. This molecule can induce apoptosis through the modulation of proteins responsible for transcriptional activation, induce cell cycle arrest [7], induce caspase-dependent apoptosis [11], and downregulate cell division proteins [12]. Additionally, GA-A has antioxidant [13] and hypolipidemic effects [14]. Different species of *Ganoderma*, such as *G. lucidum*, *G. tsugae*, *G. orbiforme*, *G. sinense*, *G. zonatum*, and *G. australe*, can produce LDTs that mediate and modulate biological systems [15]. These compounds can bind to surface receptors on immune cells, induce apoptosis, and are cytotoxic to cancerous cells, providing multiple health benefits that can aid in the treatment of chronic disease [7,15].

β-glucans are polymers composed of β-(1,3)-glucans and β-(1,6)-glucans and are an important group of bioactive compounds produced by *Ganoderma* species [7]. These polysaccharides have potential health benefits associated with immune modulation, cholesterol reduction, and cancer treatment [16]. These complex polysaccharides are present in the cell wall and fruiting bodies, mycelia, or spores and vary according to the developmental stage [7,17]. *Ganoderma* polysaccharides can promote wound healing by increasing cell migration after epithelial damage [18] and inhibit cancer growth through the induction of apoptosis, cytokine release, and immune system enhancement in vivo, among other protective effects [7]. β-glucans can modulate the immune system, complementing LDTs’ mode of action, which is directly cytotoxic to cancer cells [7]. Both LDTs and polysaccharides possess antioxidant activity, with β-glucan demonstrating a strong ability to scavenge radicals [19]. The biological activities of β-glucans make them a key contributor to the therapeutic potential of the *Ganoderma* species.

Triterpenoid and glucan contents and compositions determine the medicinal value of *Ganoderma* mushrooms. However, to the knowledge of the authors, their abundance in the Australian *Ganoderma* species has not been reported. Therefore, the morphological diversity and chemical composition characteristics of fruiting bodies from 22 Australian *Ganoderma* fruiting bodies were analyzed. Light microscopy was used to evaluate the morphologies at three distinct locations in fruiting bodies from each specimen, and the basidiospore size and shape were determined. Triterpenoids were resolved by ultrahigh-performance liquid chromatography (UHPLC) and identified using high-field Orbitrap hybrid mass spectrometry (MS). The total glucan, α-glucan, and β-glucan contents were determined using enzymatic hydrolysis. The results provide the first structural evidence of lanostene-derived triterpenoids in the Australian *Ganoderma* species.

## 2. Materials and Methods

### 2.1. Materials

Chloroform (CHCl_3_), methanol (MeOH), dimethyl sulfoxide (DMSO), sulfuric acid, hydrochloric acid, acetic acid, 2,2′-Azino-bis(3-ethylbenzothiazoline-6-sulfonic acid) (ABTS), 2,2-Diphenyl-1-picrylhydrazyl (DPPH), 2,4,6-Tris(2-pyridyl)-s-triazine (TPTZ), GA-A, gallic acid, cholic acid, agar, yeast extract, malt extract, and D-glucose were acquired from Sigma-Aldrich (St. Louis, MO, USA). Sodium hydroxide, ethanol, peptone, and HPLC-grade formic acid, water, and acetonitrile were obtained from Thermo Fisher Scientific (Waltham, MA, USA). Ferric chloride hexahydrate was supplied from Scharlau (Barcelona, Spain). Sodium acetate was obtained from VWR Chemicals (Radnor, PA, USA). GOPOD and β-glucan assay (yeast and mushroom) kits were purchased from Megazyme (Wicklow, Ireland). A powdered *G. lucidum* fruiting body extract was purchased (Teelixir, Woori Yallock, Australia); this product consisted of a dual extract (water and alcohol) of sundried, log-grown Chinese Reishi sold as a powder that contained triterpenoids and 35% (*w*/*w*) β-glucans [20].

### 2.2. Ganoderma Cultivation

Twenty-one *Ganoderma* fruiting bodies were obtained or donated from private properties across Queensland and New South Wales (Australia) between 2019 and 2022. One fruiting body (G22) was obtained by culturing *Ganoderma steyaertanum* millet grain spawn (Aussie Mushroom Supplies, Carrum Downs, Australia) in autoclaved cardboard (60% (*w*/*w*) moisture) at a 1:10 ratio at 22 °C for six weeks. Where possible, live cultures were isolated from the fruiting bodies using solid media (agar at 12 g·L^−1^, yeast extract, malt extract, glucose, and peptone at 5 g·L^−1^) by culturing tissue at 28 °C for 10 to 20 days. The leading edges of the resulting mycelia were transferred into a nutrient broth (5 g·L^−1^ of yeast extract, malt extract, glucose, and peptone) at 28 °C for 10 to 20 days. Stocks of the mycelia were prepared by mixing mycelial cultures with 10% (*v*/*v*) DMSO and stored at −80 °C. The remainder of the fruiting bodies were freeze-dried (VirTis Benchtop Pro: SP Scientific, Stone Ridge, NY, USA) and stored at −20 °C.

### 2.3. Morphological Characterisation

The abaxial and adaxial surfaces of the 22 *Ganoderma* specimens were photographed, and three distinct cross-sections per fruiting body were prepared: (i) a section perpendicular to the base of the fruiting body passing through the cutis and pore tissue, (ii) a section on the external surface parallel to the base of the fruiting body passing through the cutis, and (iii) through the pores. The cross-sections were imaged using a stereomicroscope (Leica M125, Leica Microsystems, Wetzlar, Germany) with a Leica DFC490 Digital color camera, and the images were processed using Leica Application Suite v.4.12). Automated Z-stacking was used when the sample surface was uneven.

Slices of the pore tissue from each fruiting body (~0.5 cm) were vortexed in 2 mL microcentrifuge tubes with ~1 mL of water to release the spores. Aliquots of spore suspensions (10 μL) were placed on microscope slides, covered with a rectangular coverslip (24 mm × 50 mm), and immediately analyzed under a light-field microscope (Nikon Eclipse Ni, Nikon, Minato Tokyo, Japan) with a DS-Fi2 high-definition color camera head and DS-L3 standalone control unit. Images were acquired and processed using NIS-Elements v.5.42.03). Basidiospore shape and size were expressed as averages of >10 randomly selected spores.

### 2.4. Sample Preparation and Extraction

Freeze-dried fruiting bodies were ground to a fine powder using a 50 mL grinding jar with metal ball (#1.4112, Retsch, Haan, Germany) in a cell disruptor at 30 Hz for 2 min (TissueLyser II, QIAGEN, Hilden, Germany). LDTs were extracted from the fruiting body powder using a method adapted from Ha do et al. [21]. Briefly, fruiting body powder (100 mg) was mixed with 10 mL of solvent in a glass test tube and incubated in an ultrasonic water bath (Elmasonic S 40 H, Elma, Singen, Germany) at 50 °C for 30 min. The LDT solvent was selected by extracting a mixture of G18 and G12 fruiting bodies using either DMSO, CHCl_3_, MeOH, EtOH, 75% aqueous MeOH (*v*/*v*), or 75% aqueous EtOH (*v*/*v*). Peak height and separation in subsequent UHPLC analysis indicated that 75% (*v*/*v*) MeOH was the better solvent (Supplementary Figure S1) and was used for all subsequent LDT extractions. Then, 75% (*v*/*v*) MeOH extracts in glass tubes were clarified by centrifugation at 1300 rcf and 22 °C for 5 min (Avanti J-15R, Beckman Coulter, Brea, CA, USA). A sub-sample (2 mL) of the supernatant was transferred to a clean Eppendorf tube, centrifuged at ambient temperature (22 °C) at 21,300 rcf for 5 min in a benchtop microcentrifuge (5425 R G, Eppendorf, Germany). The resulting supernatant was filtered through a 0.22 μm polypropylene hydrophilic membrane (Kinesis, Bothell, WA, USA) and stored at −20 °C. All samples were extracted in triplicate.

### 2.5. Antioxidant Activity

#### 2.5.1. ABTS Radical-Scavenging Activity

A stock solution of ABTS was prepared by mixing 2.45 mM of ammonium persulfate and 7.40 mM of ABTS. The mixture was incubated in the dark at ambient temperature for 16 h [22]. A fresh solution of ABTS was prepared daily by diluting this stock solution 1:20 in 50% aqueous methanol (*v*/*v*) to an absorbance of 1.00 ± 0.05 at 734 nm. The radical-scavenging activity of *Ganoderma* methanolic extracts was measured by mixing 300 µL of ABTS working solution with 5 µL of sample or standard in a flat-bottom 96-well plate. The mixture was incubated at room temperature for 60 min in the dark. Color removal was measured at 734 nm using a microplate reader (BioTech Synergy HTX, Agilent, Santa Clara, CA, USA). Extracts were individually diluted to fall within the activity range of gallic acid standards (0.015 to 0.15 g·L^−1^). Results were expressed as mg gallic acid equivalent/g of dry fruiting body.

#### 2.5.2. DPPH Free Radical-Scavenging Activity

A stock solution of 1.0 mM DPPH was prepared in methanol, and a fresh working solution was prepared daily by diluting the stock solution 1:5 in methanol to a final absorbance of 1.00 ± 0.05 at 517 nm. Each sample or standard solution (5 µL) was mixed with DPPH (200 µL) and incubated on ice in the dark for 60 min. Extracts were diluted to fall within the activity range of the gallic acid standards (0.015 to 0.15 g·L^−1^). The results were expressed in the form of mg gallic acid equivalent/g of the dry fruiting body.

#### 2.5.3. Ferric Reducing Antioxidant Ability (FRAP)

FRAP activity was measured according to Re et al. [22] with minor adaptations. A working solution was prepared daily by mixing 0.3 M acetate buffer with a pH of 3.6, 10 mM Fe^3+^-2,4,6-tripyridyl-s-triazine (TPTZ) in 40 mM hydrochloric acid, and 20 mM of ferric chloride 10:1:1; this solution was submerged in a water bath at 37 °C until it fully dissolved. The FRAP activity in each *Ganoderma* extract was measured by mixing 15 µL of methanolic extract with 285 µL of warm FRAP working solution. The mixture was incubated at 37 °C in the dark, and the absorbance was measured at 593 nm after 6 min. Extracts were diluted to fall within the activity range of the ferrous sulfate standards (307.3 to 15.2 g·L^−1^). Results were expressed as g ferrous sulfate equivalent/g of the dry fruiting body.

### 2.6. Glucan Quantification

Total, α-, and β-glucan contents of 19 *Ganoderma* fruiting bodies were quantified spectrophotometrically using a yeast and mushroom β-glucan assay kit (Megazyme, Wicklow, Ireland) as per the manufacturer’s instructions. The total and α-glucan contents were determined directly, while the β-glucan content was determined as the difference between the total and α-glucan contents. Briefly, freeze-dried fruiting bodies were pulverized in a 50 mL grinding jar with a grinding ball (#1.4112, Retsch, Haan, Germany) at 30 Hz for 2 min in a TissueLyser II cell disruptor (QIAGEN, Hilden, Germany). The total glucan and free D-glucose contents were quantified as follows. Ice-cold 12 M sulfuric acid (2 mL) was added to 90 mg of dried mushroom powder, mixed using a vortex, and incubated in an ice-water bath for 2 h. Water (4 mL) was added, and the tube was vortexed before an additional 6 mL of water was added. The tube was incubated in a water bath at 90 °C for 2 h, and the contents were transferred to a 100 mL volumetric flask containing 6 mL of 8.0 M NaOH. The volume was adjusted to 100 mL with 200 mM sodium acetate buffer (pH 4.5), and the contents were first mixed and then aliquoted into 1.5 mL microcentrifuge tubes. The aliquots were clarified by centrifugation in a benchtop microcentrifuge (5425 R G, Eppendorf, Germany) at 21,300 rcf for 5 min, and 0.1 mL aliquots of supernatant were digested with 0.1 mL of exo-1,3-β-glucanase (20 U·mL^−1^) plus β-glucosidase (4 U·mL^−1^) in a 200 mM sodium acetate buffer (pH 4.5) at 40 °C for 60 min. The GOPOD reagent (3 mL) was added to each tube, and the mixture was incubated at 40 °C for 20 min. The absorbance of each sample was measured at 510 nm using a benchtop spectrophotometer (Genesys 10s UV-Vis, Thermo Scientific, Waltham, MA, USA), and the total glucan concentration was determined by performing a comparison with solutions of pure glucose of a known concentration.

The α-glucan content was determined as per the manufacturer’s instructions: 100 mg of dried mushroom powder was incubated in an ice bath with 2 mL of 1.7 M NaOH for 20 min. Then, 8 mL of 1.2 M sodium acetate buffer (pH 3.8) and 0.2 mL of amyloglucosidase (1630 U·mL^−1^) plus invertase (500 U·mL^−1^) were added, and the mixture was incubated at 40 °C for 30 min in a shaker incubator at 100 rpm. After incubation, a 2 mL aliquot was clarified by centrifugation at 13,000 rpm for 5 min, and 0.1 mL aliquots of clarified supernatant were incubated with 0.1 mL of sodium acetate buffer (200 mM, pH 4.5) and 3.0 mL of GOPOD reagent. The mixture was incubated at 40 °C for 20 min, the absorbance at 510 nm was measured using a benchtop spectrophotometer (Genesys 10s UV-Vis, Thermo Scientific, Waltham, MA, USA), and the α-glucan concentration was determined by comparison to solutions of pure glucose of a known concentration. Yeast powder containing ~50% dry weight β-glucans was extracted and analyzed as a control. The difference between the total glucans and α-glucans corresponded to the β-glucan content.

### 2.7. Identification of LDTs Through UHPLC-MS/MS

LDTs were separated using a Waters Acquity UHPLC CSH C18 column (2.1 mm × 100 mm, 1.7 μm) at 30 °C in a Dionex UltiMate 3000 RSLC system (Thermo Scientific, Waltham, MA, USA) comprising a TCC-3000RS column oven, LPG-3400RS solvent pump, and a WPS-3000TRS autosampler. The injection volume was 10 μL, and the mobile phase consisted of (A) aqueous formic acid (0.1% (*v*/*v*) and (B) acetonitrile (100% (*v*/*v*)). The flow rate was 0.2 mL·min^−1^ and used the following elution profile: 0 to 6 min, 25 to 31.5% B; 6 to 8 min, 31.5 to 32% B; 8 to 28 min, 32 to 33.2% B; 28 to 36 min, 33.2 to 100% B; 36 to 43, min 100% B; and 43 to 50 min, 25% B. A diode array detector (DAD-3000, Thermo Fisher Scientific) was used to monitor absorbance at 243, 254, and 258 nm in line with a mass spectrometer (LTQ Orbitrap Elite, Thermo Scientific, Waltham, MA, USA). The mass spectrometer was equipped with an electrospray ionization source operating in negative ionization mode. Mass spectrometry was optimized using GA-A as a standard. Nitrogen was used as stealth and auxiliary gas at 36 and 13 arbitrary units, respectively. A spray voltage of 5 kV was used, and the source temperature was kept at 250 °C. Full scan mass spectra were acquired in the range of *m*/*z* 100 to 1000. A data-dependent MS/MS scan with collision-induced dissociation (CID) using 25 arbitrary units was applied to the most abundant [M–H]^−^ ion in the full scan. Tandem mass spectrometry data acquisition and analysis were performed in Xcalibur 3.0. An ANOVA analysis of the MS results was performed in GraphPad v. 9.0.0. Triplicate samples from each fruiting body were analyzed. The area under the peak of each LDT was normalized against the internal standard of cholic acid. 

Aliquots (100 μL) of cholic acid(1 mg·mL^−1^) were added per 100 mg of fruiting body powder before extraction. The method accuracy was determined by the mean percentage response of cholic acid at 1, 2, 3, 4, and 5 µg·mL^−1^ in extraction solvent-only samples. Intra-day and inter-day precision were determined by analyzing cholic acid solutions on different days and on the same day (*n* = 8). Method accuracy ranged from 1.6 to 15.4%, and intra-day and inter-day precision ranged from 1.6 to 8.1%.

## 3. Results and Discussion

### 3.1. Morphological Characterisation of Ganoderma Fruiting Bodies

**Figure 1:**
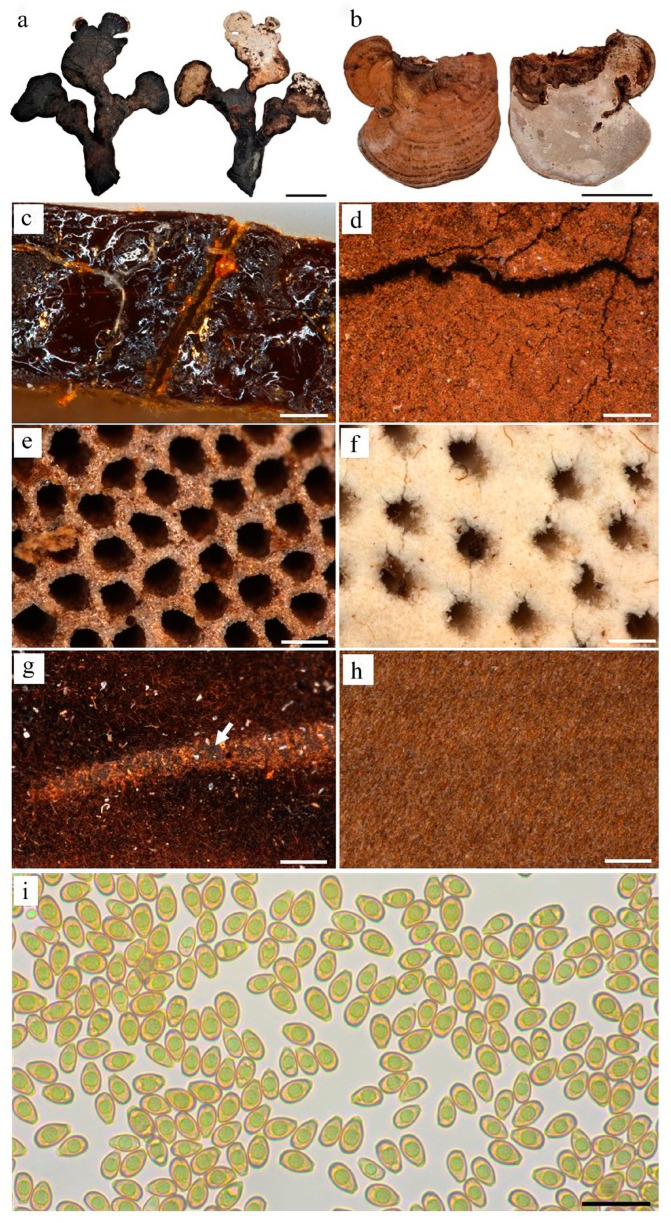
Macroscopic and microscopic morphologies of Australian *Ganoderma* spp. fruiting bodies (**a**–**i**). Abaxial and adaxial surfaces of G18 (**a**) and G2 (**b**). Pileus surfaces of laccate G17 (**c**) and matte G3 (**d**). Pore surfaces of G3 (**e**) and G10 (**f**). Context tissues of G3 (**g**) and G10 (**h**). Microscopic image of basidiospores from G5 (**i**). Arrow pointing melanoid band from G3 context tissue (**g**). Scale bars: (**a**,**b**), 5 cm; and (**c**–**h**) 200 µm; (**i**) 20 µm.

The morphologies of each pileus, pore, and stipe of a *Ganoderma* fruiting body were evaluated for size, color, and shape using fresh and freeze-dried materials (Figure 1, Supplementary Table S2). All fruiting bodies possessed concentric growth undulations on the pileus, aligning with a characteristic feature reported for *Ganoderma* fruiting bodies [23]. The presence or absence of a stipe is a critical morphological marker, distinguishing the species from the *G. lucidum* complex, which occasionally presents a stipe, to the *G. applanatum* complex, which does not present a stipe. The laccate and stipitate pileus with concentric growth undulations and characteristic double-walled basidiospores are well-documented for *G. lucidum* in the literature [23]. Out of the 22 fruiting bodies, eight had a stipe, and ten were sessile (no stipe). The four fruiting bodies that were indeterminate (received without the attachment point) were most likely sessile. Among the fruiting bodies, thirteen displayed a laccate pileus, consistent with members of the *G. lucidum* complex, while the remaining nine displayed a matte pileus, characteristic of the *G. applanatum* complex. The pore coloration varied among the specimens, with ten exhibiting cream pores—a characteristic associated with young fruiting bodies [24]. The remainder of the pores displayed shades ranging from light brown to dark brown, which was indicative of a mature fruiting body [24]. Two fruiting bodies were not morphologically assessed due to advanced degradation.

The morphological characteristics of the fruiting bodies displayed high variability, consistent with the diverse morphological features reported for *Ganoderma* species [24]. Specimen G10’s fruiting body displayed multiple deep fissures extending from the pileus to the pore tissue, a characteristic often associated with older fruiting bodies [24]. This is typical of *Ganoderma* fruiting bodies, which, unlike ephemeral medicinal mushrooms, can persist in the environment for years, depending on the species, substrate availability, and environmental conditions [25]. Another unique morphological characteristic was observed with G18. This was the darkest color specimen, and the fruiting body of specimen G18 formed pilei along an extended stipe, resembling leaves along a branch (Figure 1a). While this atypical growth could be attributed to diverse environmental conditions that impact fruiting body development [26], this morphology is indicative of *Ganoderma incrassatum* according to the Queensland Mycological Society Ganoderma fungi key. In contrast, the fruiting bodies from specimens G1, G2, G3, G4, G5, G6, G7, G14, and G21 displayed similar characteristics and were matte with medium-brown pilei, light-brown pore tissues, thin context tissues, and thick tube layers. The remaining fruiting bodies exhibited thicker context tissues and thinner tube layers. Based on the observed morphological and chemical profiles, the fruiting bodies were clustered into four groups:

*Group 1* included fruiting bodies from specimens G1 through G7, characterized by their medium-brown, matte, and rugose pileus, measuring from 9.8 to 18 cm in length and 5.7 to 11 cm in width. These fruiting bodies were sessile and imbricate. The context tissue was dark-brown and relatively thin, and the tube layer was noticeably thick. On the pore surface, a light-brown coloration was observed. These morphological features are consistent with those reported for members of the *G. applanatum* complex [27]. Basidiospores from this group were small, ranging from 9.4 to 10.5 µm in length and 5.9 to 7.5 µm in width. These dimensions are within the range reported for *Ganoderma* species in the Australasian and Pacific regions. Common LDTs found in Group 1 included **1, 3, 4, 14, 19, 23, 24, 28**, and **31**. GAs were not present in this group. On average, the fruiting bodies in this group contained 25.5 ± 3.9% (*w*/*w*) β-glucan.

*Group 2* included the fruiting bodies G8, G9, and G10, which exhibited laccate and smooth or rugose surfaces, with medium to dark-brown pilei showing shades of red and yellow. These fruiting bodies measured 6–9 cm in length and ~5 cm in width. They were sessile or stipitate, appearing imbricate, and the context tissue was medium to dark brown and thick, while the tube layer was thin. They exhibited cream to medium-brown coloration on the pore surface. Basidiospores were large, measuring 11.0 to 12.1 µm in length and 7.1 to 8.8 µm in width. Common LDTs in this group include **2, 4, 5, 8, 15, 21, 22, 26, 27,** and **30**. These fruiting bodies averaged 25.4 ± 3.8% (*w*/*w*) β-glucans.

*Group 3* consisted of fruiting bodies G11, G12, G13, G14, G15, G16, G17, G18, and G19. These fruiting bodies generally exhibited a matte pileus that was either smooth or rugose and primarily dark brown to black with shades of red. They were stipitate or sessile, and their arrangement ranged from imbricate to ungulate. The fruiting bodies ranged from 4 to 13 cm in length and 3 to 9 cm in width, with dark-brown context tissues. Basidiospores from this group were larger than those from the other groups, measuring from 9.9 to 12.3 µm in length and 4.9 to 9.9 µm in width. Compound **7** was commonly found among the fruiting bodies from this group. Group 3 exhibited the highest β-glucan content, averaging 32.5 ± 6.4% (*w*/*w*) β-glucans.

*Group 4* consisted of fruiting bodies from three specimens: G20, G21, and G22. These fruiting bodies exhibited dark-brown caps with shades of yellow, and they generally possessed laccate surfaces. They ranged from 2.4 to >3.5 cm in length and 2 to >4 cm in width. They were imbricate and could be either smooth or rugose. Their margin was cream-colored, and the context tissue and pore surfaces ranged from light to dark brown. G22 was a young fruiting body harvested before developing a tube layer, so this fruiting body could not be assessed for pore or basidiospore characteristics. The basidiospores of the remaining fruiting bodies were large, measuring from 7.0 to 12.3 µm in length and 4.4 to 8.9 µm in width. Due to the insufficient biomass of G20 and G22, only G21 was tested for β-glucan content, which was 21.1% (*w*/*w*).

The morphological diversity observed among the 22 *Ganoderma* fruiting bodies reflects the diversity levels of their species and environmental conditions. This diversity enriches the metabolic potential of these fruiting bodies since their morphologies influence their triterpenoid and polysaccharide contents, as reported by previous studies [28,29]. Our findings align with these studies, suggesting that morphological characteristics could serve as markers for selecting *Ganoderma* fruiting bodies for their distinct groups of LDTs.

### 3.2. Antioxidant Activity of Ganoderma Methanolic Extracts

The antioxidant activity of *Ganoderma* is primarily associated with the abilities of the compounds present in the fruiting body, mycelium, or spores to scavenge free radicals [30]. Antioxidant activity is often determined using the ABTS, DPPH, and FRAP assays. The choice of solvent significantly impacts these assays, as solvent polarity affects the extraction efficiency of a specific compound [31]. Methanol and ethanol are frequently reported to yield higher antioxidant activities than aqueous extracts, while ethyl acetate and chloroform generally result in lower antioxidant activities [31,32].

**Figure 2:**
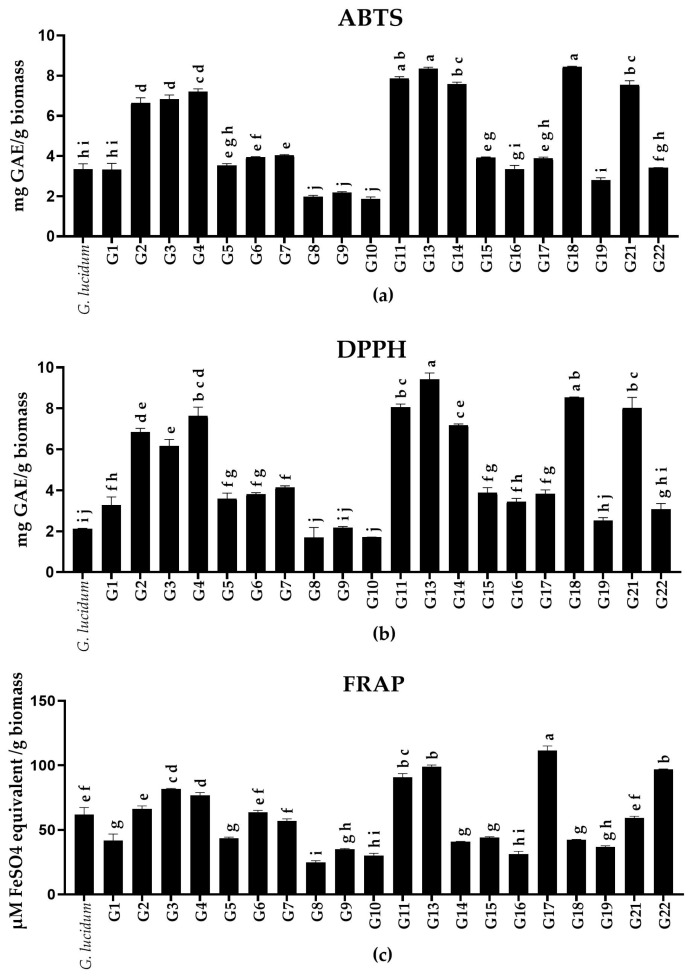
Antioxidant activities of *Ganoderma* methanolic extracts were evaluated using (**a**) ABTS and (**b**) DPPH assays. Activity is expressed as a milligram of gallic acid equivalent per gram of the dried fruiting body, and (**c**) FRAP assays are expressed as micromoles of ferrous sulfate equivalent per gram of the dried fruiting body. *G. lucidum* is a commercial product (Teelixir). Values are expressed as the mean ± SD (*n* = 2). Different lowercase letters in each bar represent statistically significant differences between fruiting bodies (*p* < 0.05) as determined by ANOVA and Tukey’s post hoc tests.

In this study, the antioxidant activities of methanolic extracts from 20 *Ganoderma* sp. fruiting bodies were assessed (insufficient biomass from G12 and G20 prevented their analysis). The highest antioxidant activities were observed in G2–4, G11, G13, and G21 (Figure 2), with values ranging from 6.6 to 8.4 mg GAE·g^−1^ DW in ABTS, 6.2 to 9.4 mg GAE·g^−1^ DW in DPPH, and 59.2 to 99.0 µmol FeSO_4_.7H_2_O·g^−1^ DW in FRAP assays. The FRAP values from this study are within the range of those reported by Dong et al. [33], who found similar antioxidant activities in *G. lucidum* extracts. The antioxidant activity of *Ganoderma* can be attributed to its peptides, polysaccharides, triterpenoids, and phenolic compounds, with peptides potentially being the main source of antioxidant activity in *G. lucidum* [32,34]. Additionally, a strong correlation between polyphenols and antioxidant activity was reported by Dong, He, Ni, Zhou, and Yang [33]. Further investigation may be necessary to assess the factors influencing antioxidant activity in *Ganoderma* species, as the production of antioxidant metabolites can be influenced by environmental factors [26,35,36].

The fruiting bodies G14 and G18 displayed relatively great ABTS and DPPH activities and FRAP antioxidant activities. The opposite occurred with G6, G17, and G22, which displayed high FRAP activities but low ABTS and DPPH activities. Pearson’s correlation coefficients were used to determine the relationships between the antioxidant activities measured by the three assays. A strong positive correlation (r = 0.99) was observed between ABTS and DPPH assays, indicating that the free radical scavenging activities measured by these methods are consistent with each other. A moderate positive correlation (r = 0.44) between ABTS/DPPH assays and FRAP assays. This suggests that, while there is a relationship between free radical scavenging activity and antioxidant power reduction, the assays are detecting distinct compounds or mechanisms of action [37]. The differences highlight the importance of employing multiple antioxidant assays for a comprehensive assessment of antioxidant capacity.

Trolox and ascorbic acid are often used as standards in ABTS and DPPH assays due to their wider concentration ranges in the calibration curve [38]. In this study, gallic acid was used as the standard due to its strong antioxidant activity and chemical stability. To enable comparisons with the literature, a conversion formula proposed by Hwang and Lee [38] was adopted. The antioxidant activity observed in this study is consistent with that reported by Zengin et al. [39], who assessed the antioxidant activity of *G. applanatum* and *G. resinaceum* aqueous and methanolic extracts. Our ABTS assay results were up to 3-fold higher than those reported by Sulkowska-Ziaja et al. [40], and the DPPH values were up to 10-fold higher than those of the same study. The distinct antioxidant capacities between these studies can be attributed to the extraction method, solvent choice, and species of *Ganoderma* [39,40,41]. Given that oxidative stress plays a major role in many illnesses, the antioxidant potential of *Ganoderma* extracts could serve as a valuable alternative in mitigating these conditions and enhancing overall health [30]. These preliminary findings may be useful to down-select some of the more potent strains that were able to be cloned prior to analysis.

### 3.3. Glucan Content in Ganoderma Fruiting Bodies

The total, α-, and β-glucan contents of Ganoderma fruiting bodies were quantified, and the data are presented in Table 1 (there is insufficient biomass for G7 and G20). The α-glucan content varied from 0.1 to 1.4% (*w*/*w*), averaging 0.3 ± 0.3% (*w*/*w*). In contrast, a high α-glucan content was observed in the commercial *G. lucidum* product (60.3% *w*/*w*), which may be attributed to the use of a bulking agent or residual substrate components, such as cereal, grains, and other starch-rich substrates [42]. Low α-glucan content in mushroom fruiting bodies is commonly described in the literature [43,44] and while this polysaccharide is essential for fungal cell wall structure and function, most of the health benefits attributed to mushrooms are linked to β-glucans [1]. The β-glucan content in *Ganoderma* fruiting bodies ranged from 19.5% (G6) to 43.5% (G18). The highest total and β-glucan contents were observed in fruiting bodies from Group 3: 35.4% (G12), 32.0% (G14), 38.3% (G15), and 36.7% (G16). No conclusions could be drawn from Group 4, as glucan content and compositional data were only available for one fruiting body from this group. The results align with those reported in the literature, where *Ganoderma* β-glucan content is described to vary significantly depending on the species and geolocation [29,45].

### 3.4. Identification of LDTs in Australian Ganoderma Fruiting Bodies

Distinguishing and characterizing LDTs using conventional analytical methods is challenging due to structural similarities and spectral overlap [15]. To overcome this limitation, an ultra-high-performance liquid chromatography-tandem mass spectrometry (UHPLC-MS/MS) method was developed to separate and identify LDTs. While the ideal identification of LDTs involves validation using pure standards, it is impractical to obtain pure compounds for over 100 known *Ganoderma* LDTs [15]. Therefore, a chromatographic method was developed to separate the compounds present in all *Ganoderma* extracts. The method was based on previous techniques used in the analysis of *Ganoderma* extracts, addressing the limitations identified in those studies. Additionally, to facilitate compound identification, a comparison against the chromatographic method developed by Li et al. [46] was employed, as the use of a similar separation column and equipment provided a compatible system for analysis. This enabled a direct comparison between our spectral data and their results, enhancing the reliability of the LDT identification present in this study. The identities of the present LDTs were further confirmed by comparing the order of elution, molecular formulae, and MS/MS fragmentation patterns with those reported in the literature [47,48,49].

**Figure 3:**
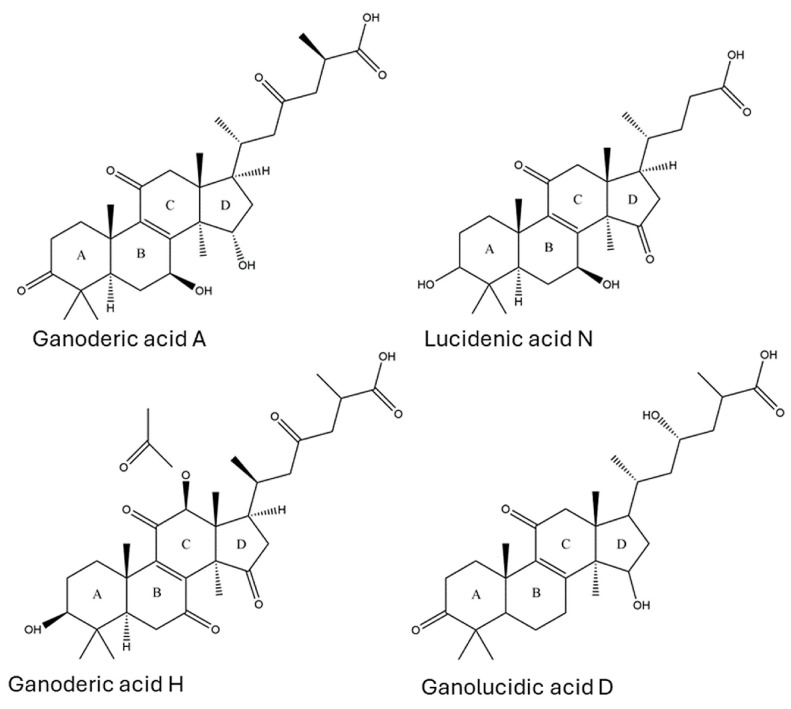
Chemical structures of four common *Ganoderma* triterpenoids: ganoderic acids, lucidenic acids, ganoderenic acids, and ganolucidic acids. These compounds share a common backbone of four rings (A–D) with varying side chains. Other triterpenoids from *Ganoderma* species have the same basic structures with minor variations in oxygen position and double bonds. Adapted from Galappaththi et al. [15].

GA-A was unequivocally identified in sample G8 with the same retention time (18.5 min) as a commercially available standard. Moreover, the observed [M-H]^−^ precursor ion (*m*/*z* 515.30, C_30_H_43_O_7_^−^) and MS/MS product ions (*m*/*z* 497.29, 453.35, 435.35, 299.19, 285.18, and 195.11) were consistent with that of GA-A, as described in the literature [46,47,50]. Although GA-A has been widely studied in *Ganoderma* species, to our knowledge, this is the first report of GA-A in the fruiting body of an Australian *Ganoderma* specimen. This suggests that Australian *Ganoderma* may share bioactive compound profiles similar to those of species from other regions. Compound **10** was observed in G19 and the *G. lucidum* extract at 13.21 min with a molecular ion of *m*/*z* 459.3125, corresponding to the chemical formula C_27_H_39_O_6_^−^. Collisional activation of this ion formed product ions at *m*/*z* 441.31, 415.33, and 385.28. Cleavage of the D-ring (Figure 3) formed diagnostic fragment ions at *m*/*z* 303.23, 289.20, 249.16, and 209.13. The retention time and precursor and product ion masses were compared against the literature, leading to the identification of compound **10** as LA-N, a cytotoxic triterpenoid [51].

**Figure 4:**
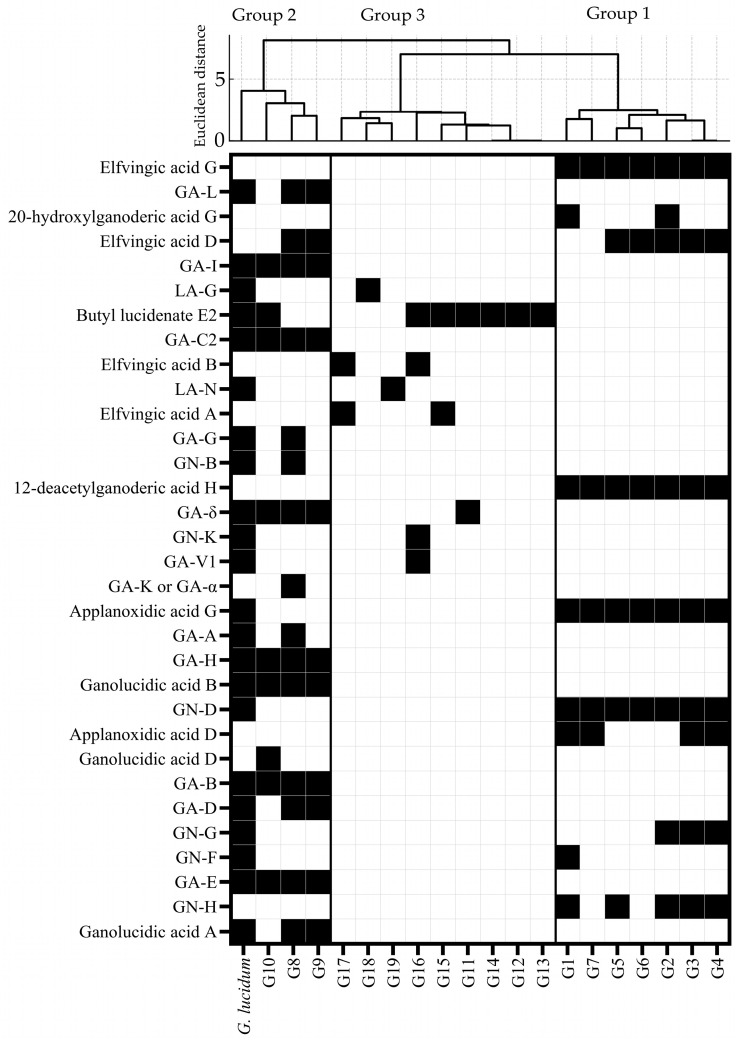
Triterpenoid distribution across 19 *Ganoderma* specimens. The heatmap indicates the presence (black) or absence (white) of each individual lanostene-derived triterpenoid (listed on the left) in *Ganoderma* fruiting bodies. Each column corresponds to a different fruiting body extract, with *G. lucidum* (first column) as a reference. The dendrogram above the heatmap clustered three Groups of fruiting bodies based on their lanostene-derived triterpenoid composition. Branches with smaller Euclidean distances indicate higher similarities. The cluster was generated using Ward’s method on Spotfire Cloud Analyst v. 14.4.0.

A total of 32 LDTs were identified in extracts from fruiting bodies of *Ganoderma* specimens using the same approach (Supplementary Table S1, Figure 4). The identified compounds include EA-G (**1**), GA-L (**2**), 20-hydroxylganoderic acid G (**3**), EA-D (**4**), GA-I (**5**), LA-G (**6**), butyl lucidenate E_2_ (**7**), GA-C2 (**8**), EA-B (**9**), LA-N (**10**), EA-A (**11**), GA-G (**12**), GN-B (**13**), 12-deacetylganoderic acid H (**14**), GA-δ (**15**), GN-K (**16**), GA-V1 (**17**), GA-K or GA-α (**18**), AA-G (**19**), GA-A (**20**), GA-H (**21**), ganolucidic acid B (**22**), GN-D (**23**), AA-D (**24**), ganolucidic acid D (**25**), GA-B (**26**), GA-D (**27**), GN-G (**28**), GN-F (**29**), GA-E (**30**), GN-H (**31**), and ganolucidic acid A (**32**). The identification of such a broad spectrum of LDTs is consistent with other analyses of *Ganoderma* triterpenoids [46,47,48,49]. No LDTs were identified in five of the *Ganoderma* specimens, suggesting that these fruiting bodies did not possess LDTs; LDTs were either present at concentrations below the limit of detection, or the LDTs therein differed significantly from those previously reported [46], thereby preventing identification.

Distinct sets of triterpenoids co-occurred within separate groups of fruiting bodies. Specifically, compounds **1, 3, 14, 19, 23, 24, 28,** and **31** were predominantly present in Group 1 specimens (G1–G7), while compounds **2, 5, 8, 15, 21, 22, 26, 27, 30,** and **32** were mainly detected in specimens from Group 2 (G8–G10). Compound **4** was present in both Groups, and its bioactivity remains unknown [52]. The primary morphological distinction between the two Groups is that Group 1 contains fruiting bodies with the matte pileus, while Group 2 contains fruiting bodies with the laccate pileus, which are important features in *Ganoderma* taxonomy [24]. The correlations between chemical composition and morphological characteristics as a means to aid taxonomic identification, termed morpho-chemotaxonomy, could significantly benefit the taxonomic identification within the chaotic *Ganoderma* genus. However, the lack of comprehensive morphological description in many studies hinders the establishment of this relationship [53].

**Figure 5:**
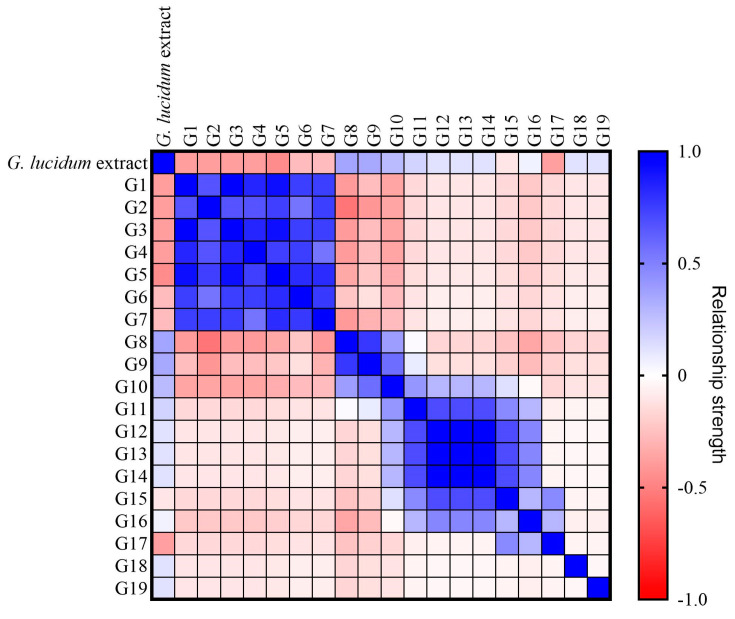
Spearman’s r correlation matrix indicates the relationship between 19 *Ganoderma* fruiting bodies based on the composition of extract lanostene-derived triterpenoids. The relationship strength and direction are indicated in the color gradient, where positive monotopic correlations are represented in blue (+1 value), negative relationships are indicated in red (−1 value), and the absence of a relationship is represented in white (0 value).

The correlation between LDT content and composition in similar fruiting bodies allowed the specimens to be divided into 4 groups (Figure 5); two of them are described above. The third group consists of fruiting bodies containing few LDTs (1 to 4 LDTs). This group comprises G11, G12, G13, G14, G15, G16, G17, G18, and G19 fruiting bodies. Finally, the fourth group comprises fruiting bodies from which no LDTs could be identified: G20, G21, and G22. The observed chemical and morphological clustering suggests that the chemical constituents of *Ganoderma* are associated with their morphology or taxonomy. However, due to the complexity of *Ganoderma* taxonomy and morphology, this relationship is not fully understood.

Compounds **1, 14, 19,** and **23** occurred in all members of Group 1; the highest contents of **1** and **14** occurred in G6 and G2, respectively, and the highest contents of **19** and **23** were both observed in G4. While the biological effects of compounds **1** and **14** were unknown, **19** (AA-G) could inhibit tumor promoters in Epstein–Barr infection [54], and **23** (GN-D) had anti-tumor effects in vivo in cervical carcinoma [55]. Differences in LDT concentration within a group could be related to factors such as genetic composition, developmental stage, and environmental conditions during fruiting body formation [26]. Group 2 shared 7 common LDTs: **5, 8, 15, 21, 22, 26,** and **30**. Most of these compounds were found at relatively high contents in G8 (**5, 8, 15,** and **21**), while **22, 26,** and **30** were more abundant in G9. Multiple health benefits are associated with these compounds, such as anti-aging (**8, 26**), hepatoprotective, neuroprotective (**30**), antioxidative (**26**), anti-hypertensive (**21, 26, 27**), anti-HIV (**21, 26**), and anti-tumor (**21, 26, 30**) effects in vitro and in vivo [12,51,56,57,58]. Specimen G8 exhibited the highest total LDT content and the greatest variety of LDTs among the fruiting bodies analyzed. In addition, G8 contained **20** (GA-A), the most well-studied LDT in *Ganoderma* due to its diverse biological activities, including anti-protease activity against HIV, promoting neuroprotection during oxidative stress in vitro [59], and anti-tumor activity [58,60]. G8 shared the largest number of LDTs (12) with the medicinal *G. lucidum* extract. Due to the high contents of multiple bioactive LDTs and its similarity with *G. lucidum* extract, specimen G8 is the *Ganoderma* of the most therapeutic value identified in this study. Group 3 contained a small number of LDTs, with **7** (butyl lucidenate E_2_) being prevalent among these fruiting bodies [61]. Compound **7** has been reported to cause mild cytotoxicity to leukemia virus-transformed cell lines, suggesting mild antitumor activity. The highest contents of this compound were detected in G12 and G13. No LDTs were identified in samples of fruiting bodies from Group 4. The absence of LDTs in this group could be the result of these compounds being present in concentrations below the method’s detection limit or the presence of novel LDTs, which is not covered by the current identification method.

The LDTs identified in this study have been associated with multiple desirable biological effects. Oxidative deterioration can cause cellular and tissue damage, inflammation, aging, and increased risk of chronic diseases [28]. To prevent such disorders, natural antioxidants, such as compound **19** (AA-G), can potentially protect cells against oxidative stress. This compound is found in all members of Group 1. Breast cancer is a malignant disease characterized by the uncontrolled growth of abnormal cells in the breast tissue. Compound **21** (GA-H) is a potential therapeutic agent capable of inhibiting transcription factors associated with cancer invasion, proliferation, and metastasis and reducing the secretion of a protease associated with cell adhesion and migration [12]. These compounds were observed in all members of Group 2 and most abundantly in specimen G8. Finally, chronic inflammation can cause destructive effects on the human body, such as tissue damage, chronic pain, cardiovascular diseases, neurodegenerative disorders, and cancer [62]. The administration of natural anti-inflammatory agents, such as compound **7** (Butyl lucidenate E_2_), can aid in the treatment and prevention of such undesirable effects. This compound can be found in most members of Group 3. The presence of bioactive LDTs in Australian *Ganoderma* specimens suggests potential therapeutic applications as they display antioxidative, anti-tumor, and anti-inflammatory properties. These findings are consistent with in vitro and in vivo studies that assess the pharmacological activities of *Ganoderma* LDTs [7,8,15]. Finally, since various LDTs possess different biological effects, fruiting bodies from groups 1, 2, and 3 can exhibit different therapeutic potential, and products for specific diseases can be prepared from their fruiting bodies.

## 4. Conclusions

The morphologies of twenty-one wild and one cultivated Australian *Ganoderma* fruiting bodies were evaluated. Specimens were assigned to four groups based on their morphologies. Their triterpenoid and glucan contents and compositions, as well as their antioxidant activities, were determined. The data revealed significant morphological and chemical diversities, with notably varied antioxidant capacities among all groups of fruiting bodies. A significant range in β-glucan content was observed, with Group 3 possessing the highest β-glucan content. Group 2 exhibited the lowest antioxidant activity but presented the highest triterpenoid similarity with the bioactive *G. lucidum*. Laccate fruiting bodies were more frequent among the specimens and presented the highest number of lanostene-derived triterpenoid (LDT), including multiple ganoderic acids—a group of compounds originally identified in *G. lucidum*. In contrast, matte fruiting bodies contained multiple ganoderenic, applanoxidic, and elfvingic acids, not ganoderic acids. The LDTs identified in this study have been associated with multiple beneficial biological effects in vitro and in vivo. The fruiting body G8 (Group 2) was the most relevant specimen in this study as it possessed a wider variety and relative abundance of bioactive LDTs, including the bioactive ganoderic acid A. The medicinal value of this specimen, as well as the chemically similar specimens G9 and G10, should be further investigated as a potential substitute for *G. lucidum* for commercial cultivation in Australia. The antioxidant activities observed and the presence of bioactive LDTs and glucans in Australian *Ganoderma* highlight their potential therapeutic application. These results represent the first formal study to identify key triterpenoids in *Ganoderma* and represent a notable step towards unlocking the medicinal potential of *Ganoderma* species in Australia. Further research should not only focus on the isolation of triterpenoids and polysaccharides for elucidation of structure and individual biological activity but also seek to culture fruiting bodies under controlled conditions to determine factors that impact triterpenoid and glucan synthesis. This research provides a foundation for exploring Australian *Ganoderma* species as valuable contributors to complementary medicine and therapeutic applications.

## Figures and Tables

**Table 1:** Polysaccharide content in *Ganoderma* fruiting bodies. The total glucan content is expressed as mg per gram of dry weight (DW), while the α-glucan and β-glucan are expressed as percentages of total glucan. Superscript letters next to the total glucan values indicate significant differences between samples, determined by ANOVA. Values with the same letter are not significantly different (*p* > 0.1).

	Sample ID	Total Glucan (mg/g DW)	α-Glucan (%)	β-Glucan (%)
	*G. lucidum* extract	619.2 ^a^	97.4	2.6
Group 1	G1	237.3 ^gi^	0.8	99.2
	G2	300.2 ^ef^	0.7	99.3
	G3	293.0 ^eg^	0.7	99.3
	G4	240.4 ^gi^	0.8	99.2
	G5	272.6 ^fg^	0.7	99.3
	G6	198.2 ^i^	1.5	98.5
Group 2	G8	215.0 ^hi^	0.5	99.5
	G9	301.4 ^ef^	3.3	96.7
	G10	270.4 ^fg^	5.2	94.8
Group 3	G11	285.8 ^eg^	0.7	99.3
	G12	359.6 ^cd^	0.8	99.2
	G13	294.9 ^ef^	1.0	99.0
	G14	321.2 ^de^	0.6	99.4
	G15	385.3 ^bc^	0.5	99.5
	G16	368.0 ^cd^	0.3	99.7
	G17	301.1 ^eg^	0.7	99.3
	G18	443.5 ^b^	0.7	99.3
	G19	299.9 ^ef^	0.7	99.3
Group 4	G21	214.0 ^fgh^	1.4	98.6
	G22	210.6 ^hi^	1.5	98.5
	G23	270.9 ^eg^	0.8	99.2

## Data Availability

The original contributions presented in the study are included in the article/Supplementary Materials, further inquiries can be directed to the corresponding author.

## Supplementary Materials

The following supporting information can be downloaded at https://www.mdpi.com/article/10.3390/jof10100723/s1, Figure S1: Peak areas of six lanostene-derived triterpenoids in the mass spectrum of samples extracted using five different solvents (*n* = 3); Table S1: Major fragments of 32 LDTs identified in Australian *Ganoderma* species; Table S2: Morphological description of 22 *Ganoderma* fruiting bodies.
