# Supplementary material for: Distributions of Lanostene-Derived Triterpenoids and Glucan Content in the Fruiting Bodies of the Australian Ganoderma Species
Source: J Fungi (Basel). 2024 Oct 18;10(10):723. doi: 10.3390/jof10100723 (PMC11509325; doi:10.3390/jof10100723)
Supplement: Supplementary file 1 [file jof-10-00723-s001.zip › jof-3249556-supplementary.pdf]

Supplementary material

Distribution of lanostene-derived triterpenoids and glucan content in the fruiting bodies of Australian *Ganoderma* spp.

Aline D. O. Campos [1,2], Mark D. Harrison [1,3], David L. Marshall [4], and P. James Strong [1,2]*

[1] Centre for Agriculture and the Bioeconomy, Queensland University of Technology, Brisbane, Australia

[2] School of Biology and Environmental Science, Queensland University of Technology, Brisbane, Australia

[3] School of Mechanical, Medical, and Process Engineering, Queensland University of Technology, Brisbane, Australia

[4] Central Analytical Research Facility, Queensland University of Technology, Brisbane, Queensland, Australia

* To whom correspondence should be addressed:

James Strong, pjstrong@gmail.com

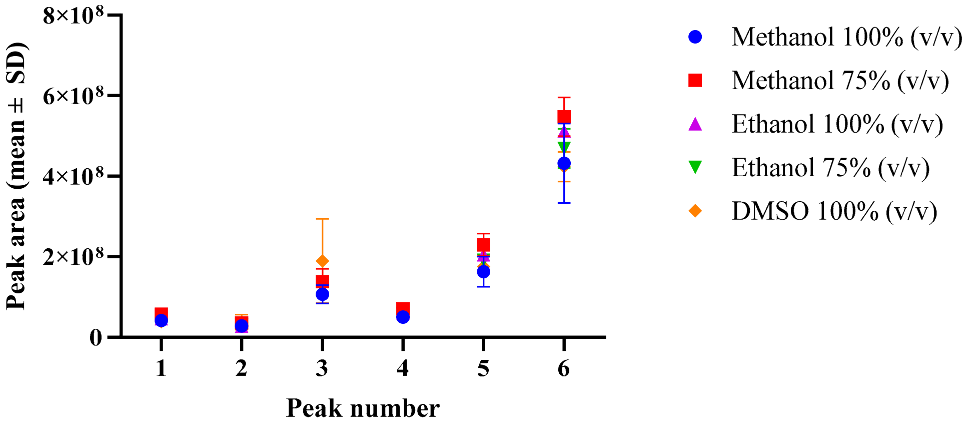

Figure S1. Peak area of six lanostene-derived triterpenoid-derivates in the mass spectrum of samples extracted using five different solvents (n = 3). Peak areas are expressed as mean ± SD. The peak areas of LDTs in 75% (v/v) methanol extracts were significantly greater (ANOVA, $p<0.0208$) than those in extracts prepared using other solvents

Table S1. Major fragments of 32 lanostene-derived triterpenoids identified in Australian *Ganoderma* species.

Peak number	RT (min)	Observed *m/z* $[M-H]^-$	Formula	Tentative identification	Major fragments and their relative intensity
**1**	6.50	545.2746	$C_{30}H_{42}O_9$	EA-G	527.29 (99.1), 517.33 (5.69), 515.40 (6.9), 513.40 (7.3), 509.37 (7.1), 501.26 (100.0), 500.43 (13.4), 499.26 (7.3), 497.39 (10.2), 483.32 (26.3), 477.12 (7.5), 473.28 (5.4), 465.38 (6.0), 457.25 (26.4), 455.27 (5.6), 439.35 (11.4), 415.265 (89.78)
**2**	6.79	533.3107	$C_{30}H_{46}O_8$	GA-L	515.35 (100), 497.3 (3.4), 485.3 (12.5), 475.4 (0.4), 471.4 (3.9), 467.3 (3.2), 455.3 (1.0), 453.4 (2.9), 449.4 (0.6), 441.4 (0.7), 423.4 (1.3), 411.3 (0.4), 405.4 (0.5), 319.3 (3.7), 318.2 (3.0), 317.2 (3.6), 303.2 (3.3), 301.3 (0.3), 195.2 (0.8)
**3**	7.26	547.2902	$C_{30}H_{44}O_9$	20-hydroxylganoderic acid G	529.29 (1.6), 503.33 (1.1), 501.38 (0.6), 485.30 (0.4), 478.95 (0.2), 419.26 (0.3), 417.91 (1.6), 417.25 (100), 416.52 (0.6), 399.30 (5.6), 389.35 (1.4), 381.29 (3.0), 373.38 (0.3), 371.37 (0.3), 359.33 (0.9), 303.24 (1.6), 265.20 (0.3)
**4**	7.47	545.2746	$C_{30}H_{42}O_9$	EA-D	527.34 (16.6), 517.24 (1.1), 515.33 (2.3), 509.33 (1.5), 501.27 (17.1), 500.44 (0.9), 499.31 (1.9), 497.32 (1.5), 483.32 (6.0), 477.02 (4.2), 473.38 (2.0), 471.20 (3.9), 465.51 (1.2), 457.13 (6.0), 455.42 (1.9), 439.10 (1.0), 415.29 (100), 399.39 (0.9)
**5**	9.50	531.2949	$C_{30}H_{44}O_8$	GA-I	516.3 (20.5), 513.33 (99.6), 498.34 (7.1), 495.34 (5.3), 489.29 (22.9), 487.35 (11.3), 471.30 (6.1), 469.34 (20.5), 457.30 (100), 454.29 (5.5), 451.39 (3.9), 443.32 (6.9), 442.30 (9.9), 439.31 (13.9), 427.30 (10.9), 399.34 (4.6), 305.23 (5.6), 303.23 (4.3), 287.23 (4.1), 265.20 (11.0)
**6**	11.15	475.269	$C_{27}H_{40}O_7$	LA-G	461.55 (0.1), 460.44 (0.3), 459.49 (0.1), 458.51 (0.2), 457.29 (100.0), 445.32 (0.2), 442.35 (0.1), 439.28 (1.0), 431.42 (0.2), 429.29 (0.2), 427.34 (0.1), 413.31 (0.2), 401.35 (0.1), 395.38 (0.3), 369.34 (0.1), 305.22 (1.2), 303.25 (0.2), 287.24 (0.2), 193.20 (0.1)
**7**	11.74	571.2896	$C_{32}H_{44}O_9$	Butyl lucidenate E_2	553.28 (4.5), 541.23 (1.3), 530.28 (1.9), 529.29 (100.0), 527.34 (1.7), 511.29 (3.9), 510.41 (0.7), 509.31 (19.5), 499.26 (2.4), 497.99 (2.8), 497.26 (84.5), 483.31 (1.0), 467.28 (7.0), 456.32 (1.2), 455.27 (63.1), 440.31 (0.4), 425.26 (1.8), 411.35 (0.5), 303.26 (1.6)
**8**	11.88	517.3159	$C_{30}H_{46}O_7$	GA-C_2	500.63 (0.4), 499.31 (100.0), 481.36 (4.5), 473.51 (0.2), 455.34 (6.2), 439.34 (0.9), 437.33 (7.5), 395.28 (0.4), 385.31 (0.4), 377.29 (0.3), 303.20 (5.5), 302.17 (4.2), 301.18 (4.2), 287.17 (7.0), 285.24 (0.5), 267.26 (0.2), 197.15 (0.5), 195.17 (1.4), 179.05 (0.2)
**9**	11.96	527.2645	$C_{30}H_{40}O_8$	EA-B	509.32 (2.6), 497.31 (100.0), 483.35 (5.8), 479.31 (12.0), 467.34 (2.0), 465.33 (3.0), 454.31 (13.9), 453.33 (40.4), 449.30 (12.9), 435.32 (22.5), 423.30 (16.9), 417.32 (8.4), 405.33 (3.8), 369.29 (2.4), 325.27 (2.6), 317.26 (2.9), 315.21 (2.3), 287.21 (7.2), 285.20 (14.4), 257.23 (2.1)
**10**	13.20	459.2745	$C_{27}H_{40}O_6$	LA-N	444.31 (14.7), 442.32 (4.8), 441.32 (100.0), 429.27 (4.4), 423.32 (24.6), 415.34 (9.2), 399.35 (5.1), 397.32 (44.6), 385.26 (36.5), 379.29 (9.3), 331.26 (15.1), 303.24 (10.1), 289.20 (37.8), 287.22 (23.5), 263.19 (32.4), 259.20 (28.9), 249.15 (36.8), 209.13 (23.1), 195.09 (12.3)
**11**	13.31	527.2642	$C_{30}H_{40}O_8$	EA-A	509.30 (9.6), 497.32 (6.0), 491.31 (6.4), 483.33 (6.6), 465.32 (27.2), 453.29 (100.0), 447.34 (1.7), 435.29 (5.7), 423.28 (2.0), 420.26 (2.0), 417.29 (1.1), 405.29 (0.9), 401.30 (0.9), 385.32 (0.6), 351.32 (0.6), 339.26 (0.6), 317.27 (0.8), 299.24 (0.7), 289.22 (0.6)
**12**	13.90	531.29492	$C_{30}H_{44}O_8$	GA-G	[513.28] 498.23 (6.9), 495.32 (100.0), 469.34 (100.0), 454.29 (34.0), 451.33 (27.7), 439.34 (1.6), 436.29 (14.3), 423.35 (1.8), 321.28 (2.4), 319.21 (6.9), 305.24 (2.1), 303.24 (10.1), 302.22 (4.6), 301.22 (16.1), 287.19 (4.5), 265.17 (44.8), 249.18 (6.2), 231.21 (1.8), 207.21 (1.7), 205.21 (1.7)
**13**	13.98	513.2845	$C_{30}H_{42}O_7$	GN-B	498.27 (0.3), 497.22 (0.2), 496.15 (1.8), 495.25 (44.6), 469.34 (0.9), 454.36 (0.3), 453.61 (0.2), 451.98 (2.6), 451.31 (100.0), 439.42 (0.2), 437.12 (0.2), 436.29 (27.3), 433.39 (1.0), 423.34 (0.4), 303.27 (0.5), 302.26 (1.3), 301.26 (0.6), 287.23 (0.3), 265.24 (0.4), 249.27 (0.3)
**14**	14.17	529.2804	$C_{30}H_{42}O_8$	12-deacetylganoderic acid H	530.37 (4.5), 529.39 (100.0), 528.45 (3.2), 511.39 (40.8), 499.34 (18.0), 493.45 (2.8), 485.37 (7.0), 481.37 (6.9), 478.43 (2.8), 467.47 (8.9), 463.26 (1.8), 449.40 (4.0), 441.36 (1.6), 419.38 (1.8), 415.39 (2.0), 399.35 (21.8), 397.32 (1.8), 379.47 (2.3), 317.32 (2.9), 301.30 (3.0)

Peak number	RT (min)	Observed *m/z* [M-H]-	Formula	Tentative identification	Major fragments and their relative intensity
**15**	14.89	515.30013	$C_{30}H_{44}O_7$	GA-δ	497.38 (0.5), 482.28 (0.7), 479.34 (1.1), 454.00 (1.8), 453.35 (100.0), 438.31 (6.6), 435.35 (1.8), 420.36 (1.1), 409.37 (4.9), 303.24 (26.0), 302.26 (2.6), 301.24 (1.0), 289.23 (1.1), 288.29 (0.3), 287.22 (11.6), 285.22 (5.6), 263.22 (0.6), 249.17 (11.5), 195.19 (1.3), 189.20 (0.4)
**16**	15.93	571.2897	$C_{32}H_{44}O_9$	GN-K	557.39 (0.5), 556.15 (0.9), 555.33 (0.9), 553.99 (3.8), 553.34 (100.0), 542.05 (0.5), 539.42 (0.4), 535.42 (1.8), 534.38 (3.1), 533.37 (3.0), 530.47 (1.1), 529.41 (25.6), 527.72 (0.7), 525.32 (0.4), 513.39 (1.5), 507.58 (0.4), 466.35 (0.4), 458.35 (0.7), 303.70 (0.7)
**17**	16.29	513.2847	$C_{30}H_{42}O_7$	GA-V1	498.28 (3.3), 495.30 (19.6), 479.32 (4.9), 477.33 (2.1), 469.34 (7.4), 462.27 (2.6), 451.33 (10.1), 447.33 (1.2), 439.30 (100.0), 433.34 (7.5), 424.29 (4.3), 381.27 (8.7), 301.25 (8.2), 299.25 (1.6), 287.22 (2.7), 285.22 (1.9), 247.21 (1.4), 225.17 (2.0), 193.10 (15.5)
**18**	16.63	573.30591	$C_{32}H_{46}O_9$	GA-K or GA-α	555.31 (80.2), 540.26 (4.9), 537.34 (1.1), 513.31 (18.7), 511.33 (100.0), 496.32 (26.2), 495.33 (6.1), 480.32 (1.5), 478.31 (4.4), 469.33 (94.7), 451.33 (34.3), 433.36 (1.8), 303.22 (12.5), 302.22 (26.1), 301.22 (4.1), 287.20 (2.3), 285.26 (2.2), 284.25 (1.1), 265.15 (8.4)
**19**	17.57	527.2637	$C_{30}H_{40}O_8$	AA-G	511.03 (0.4), 510.11 (1.6), 509.24 (100.0), 508.56 (1.2), 507.48 (0.5), 499.42 (0.4), 483.33 (1.4), 482.10 (0.4), 481.29 (22.6), 466.26 (0.4), 465.29 (32.3), 459.33 (8.0), 453.39 (0.4), 439.40 (0.6), 437.30 (4.1), 421.46 (0.8), 355.32 (0.5), 343.41 (0.5), 317.23 (2.6)
**20**	18.50	515.3001	$C_{30}H_{44}O_7$	GA-A	497.33 (100.0), 485.35 (0.8), 479.32 (4.8), 471.34 (0.5), 453.33 (6.6), 447.01 (0.4), 437.35 (0.7), 435.34 (8.5), 423.28 (0.4), 417.35 (0.3), 399.36 (0.3), 393.33 (0.3), 355.32 (0.4), 301.22 (8.2), 300.2 (5.2), 299.19 (6.5), 285.21 (4.7), 283.23 (0.4), 195.15 (1.8)
**21**	18.52	571.2898	$C_{32}H_{44}O_9$	GA-H	556.01 (0.4), 555.15 (0.3), 554.23 (1.4), 553.32 (100.0), 536.34 (0.6), 535.34 (10.1), 534.36 (1.0), 533.35 (2.0), 530.44 (0.5), 529.36 (9.1), 527.55 (0.3), 527.0 (0.6), 526.19 (0.3), 525.37 (1.6), 513.38 (0.8), 511.46 (0.5), 502.97 (0.3)
**22**	18.67	501.3209	$C_{30}H_{46}O_6$	Ganolucidic acid B	484.40 (3.6), 483.38 (62.7), 482.53 (2.1), 465.38 (1.1), 440.39 (4.4), 439.41 (100.0), 423.37 (2.8), 421.42 (3.2), 290.25 (0.9), 289.26 (28.9), 288.24 (1.3), 287.26 (41.0), 273.25 (1.4), 194.20 (1.3), 193.21 (1.3), 179.16 (0.9), 149.12 (2.4)
**23**	18.96	529.27922	$C_{30}H_{40}O_7$	GN-D	496.27 (20.1), 493.31 (24.5), 478.32 (2.3), 467.32 (100.0), 452.31 (46.3), 449.32 (59.7), 434.29 (14.7), 431.33 (3.2), 421.38 (2.7), 319.25 (3.8), 317.22 (9.5), 303.24 (3.4), 301.22 (14.0), 300.22 (8.3), 299.21 (18.8), 285.20 (10.0), 263.16 (73.0), 249.19 (9.9), 231.21 (2.7), 205.16 (3.8)
**24**	20.44	527.2644	$C_{30}H_{40}O_8$	AA-D	509.36 (1.0), 497.29 (23.9), 491.4 (0.3), 483.36 (0.6), 479.27 (9.9), 465.36 (2.0), 447.36 (0.7), 439.28 (2.3), 413.34 (0.8), 395.32 (0.7), 383.32 (0.7), 379.32 (0.3), 377.31 (1.3), 365.27 (0.9), 355.28 (0.4), 353.29 (0.3), 315.23 (0.6), 289.23 (0.5)
**25**	20.54	499.3061	$C_{30}H_{44}O_6$	Ganolucidic acid D	481.36 (1.2), 456.08 (1.0), 455.37 (100.0), 453.42 (0.4), 437.41 (1.2), 411.43 (0.4), 385.31 (23.5), 367.31 (0.4), 357.32 (1.8), 343.32 (0.4), 325.28 (6.1), 323.3 (0.9), 313.33 (0.4), 295.3 (0.6), 287.24 (6.1), 285.26 (1.5), 269.3 (0.3), 259.27 (0.3)
**26**	20.92	515.300	$C_{30}H_{44}O_7$	GA-B	497.35 (100.0), 479.43 (4.5), 471.41 (4.5), 453.36 (53.5), 437.33 (20.7), 435.37 (21.9), 425.41 (4.9), 417.29 (20.6), 399.31 (6.1), 393.35 (7.4), 377.24 (8.1), 355.29 (10.7), 315.29 (5.0), 301.22 (17.4), 300.25 (3.5), 299.32 (4.0), 297.28 (4.8), 285.21 (22.7), 193.12 (3.6)
**27**	24.27	513.28435	$C_{30}H_{42}O_7$	GA-D	[495.34] 480.31 (1.2), 477.29 (1.8), 451.32 (100.0), 436.28 (8.2), 433.33 (2.0), 418.32 (1.2), 407.37 (3.9), 301.21 (27.7), 300.23 (3.5), 299.27 (1.0), 287.23 (1.6), 285.19 (11.2), 283.19 (7.7), 268.25 (0.5), 261.19 (0.6), 247.16 (11.2), 227.21 (0.4), 193.19 (1.2), 149.07 (2.6)
**28**	25.15	511.2687	$C_{30}H_{40}O_7$	GN-G	493.29 (100.0), 478.32 (1.0), 475.28 (16.2), 453.26 (4.7), 450.08 (1.5), 449.30 (58.0), 435.28 (1.4), 431.31 (18.0), 416.34 (1.5), 409.37 (1.3), 405.40 (1.4), 329.23 (4.9), 327.20 (16.1), 287.18 (14.0), 286.19 (5.7), 285.17 (49.2), 269.22 (1.1), 207.16 (2.1), 183.16 (1.1), 181.14 (2.1)

Peak number	RT (min)	Observed *m/z* [M-H]$^-$	Formula	Tentative identification	Major fragments and their relative intensity
**29**	25.22	509.253	$C_{30}H_{38}O_7$	GN-F	494.23 (0.6), 492.65 (0.5), 491.25 (100.0), 479.24 (0.3), 476.23 (8.1), 473.31 (0.7), 465.99 (0.7), 465.28 (22.6), 461.94 (1.2), 461.22 (66.2), 448.10 (0.8), 447.27 (42.3), 441.09 (0.8), 432.27 (3.2), 429.33 (0.4), 417.32 (1.0), 301.21 (1.2), 300.23 (1.2), 299.22 (2.9)
**30**	26.99	511.2688	$C_{30}H_{40}O_7$	GA-E	493.26 (65.0), 469.31 (0.2), 467.35 (2.6), 454.93 (0.2), 452.31 (0.3), 451.16 (0.4), 450.08 (1.2), 449.31 (100.0), 435.17 (0.2), 434.3 (14.7), 431.34 (0.8), 421.37 (0.4), 301.28 (0.5), 300.24 (1.3), 299.27 (0.4), 285.27 (0.2), 247.22 (0.3)
**31**	29.16	511.2692	$C_{30}H_{40}O_7$	GN-H	493.32 (43.5), 481.32 (1.9), 478.32 (8.2), 467.25 (40.5), 463.33 (2.2), 450.14 (1.2), 449.37 (100.0), 439.37 (3.1), 431.36 (2.8), 405.44 (1.3), 401.20 (3.8), 397.33 (2.9), 381.29 (1.3), 379.36 (1.7), 359.21 (3.3), 317.28 (2.2), 315.22 (2.7)
**32**	29.78	499.3052	$C_{30}H_{44}O_6$	Ganolucidic acid A	482.39 (2.4), 481.35 (42.3), 463.37 (0.6), 455.42 (0.5), 438.37 (4.0), 437.4 (100.0), 421.39 (1.3), 419.42 (2.8), 393.43 (0.5), 288.28 (0.5), 287.25 (16.9), 286.29 (0.8), 285.24 (29.5), 269.26 (0.9), 195.20 (0.8), 194.18 (0.9), 193.15 (0.9), 149.16 (1.1)

Table S2. Morphological description of 22 *Ganoderma* fruiting bodies

Sample ID	Description
**G1**	Basidiocarp sessile, laterally attached. Pileus surface medium-brown, matte, 9.5 cm in length, 5.7 cm wide, and 1.2 cm thick at the base, dimidiate. Pileus imbricate, rugose, with concentric undulations and fissures. Regular cream margin. Context dark brown, spongy texture, 0.5 cm thick, and present melanoid band. Tube layer dark-brown, 0.8 cm thick. Pore surface light-brown, 157.75 mm x 123.75 mm, 5 (5: 6) pores per mm, 62 mm between pores, and 194 mm between pore axes. Basidiospores ovate, 7.47 – 10.17 µm x 4.41 – 5.71 µm, truncate.
**G2**	Basidiocarp sessile, laterally attached. Pileus surface medium-brown, matte, 10 cm in length, 9 cm wide, and 2.5 – 3 cm thick at the base, box-shaped. Pileus imbricate, rugose, with concentric undulations and fissures. Regular medium-brown margin. Context dark brown, spongy texture, 1.7 – 0.4 cm thick, and absent melanoid band. Tube layer dark-brown, 1.1 cm thick. Pore surface light-brown, round pores, 106.8 mm x 113.1 mm, 6 (5: 7) pores per mm, 101.42 mm between pores, and 203.88 mm between pore axes. Basidiospores ovate, 6.56 – 9.86 µm x 4.8 – 8.49 µm, truncate.
**G3**	Basidiocarp sessile, laterally attached. Pileus surface medium-brown, matte, 10 cm in length, 6.5 cm wide, and 1.5 thick at the base, reniform. Pileus imbricate, rugose, with concentric undulations and fissures. Regular light-brown margin. Context dark brown, spongy texture, 0.4 cm thick, and present melanoid band. Tube layer dark brown, becoming lighter near the outer edge, 0.1 – 1 cm thick. Pore surface light-brown, round pores, 61.07 mm x 58.96 mm, 8 (8: 9) pores per mm, 39.23 mm between pores, and 126.66 mm between pore axes. Basidiospores ovate, 7.1 – 9.76 µm x 4.96 – 6.07 µm, truncate.
**G4**	Basidiocarp sessile, laterally attached. Pileus surface medium-brown, matte, 12 cm in length, 9 cm wide, and 1.5 cm thick at the base, suborbicular. Pileus imbricate, rugose, with concentric undulations and fissures. Irregular light-brown margin. Context dark brown, spongy texture, 0.7 cm thick, and present melanoid band. Tube layer dark-brown, 1 cm thick. Pore surface light-brown, pores round, slightly oval, 104.42 mm x 86.84 mm, 6 (5: 6) pores per mm, 75.6 mm between pores, and 181.13 mm between pore axes. Basidiospores ovate, 6.93 – 9.68 µm x 4.95 – 5.62 µm, truncate.
**G5**	Basidiocarp sessile, laterally attached. Pileus surface medium-brown, matte, 18 cm in length, 11 cm wide, and 0.5 cm thick at edge, dimidiate. Pileus imbricate, rugose, with concentric undulations and fissures. Irregular cream margin. Context dark brown, spongy texture near the bottom and woody near the top, 0.8 cm thick, and absent melanoid band. Tube layer medium-brown, 0.7 cm thick. Pore surface light-brown, slightly oval, 150 mm x 135.64 mm, 5 pores per mm, 77.93 mm between pores, and 206.73 mm between pore axes. Basidiospores obovate, 7.31 – 9.38 µm x 4.94 – 6.24 µm, truncate.
**G6**	Basidiocarp surface medium-brown, matte, and 0.8 cm thick at the edge, with concentric undulations and fissures, light-brown margin. Context dark brown, spongy texture near the bottom and woody near the top, 0.7 cm thick, and absent melanoid band. Tube layer dark-brown, 1 – 6 cm thick with clear white strikes near the outer edge. Pore surface light-brown, pores oval, 141.07 mm, 6 (5: 6) pores per mm, 49.11 mm between pores, and 235.3 mm between pore axes. Basidiospores ovate, 5.92 – 10.45 µm x 5.08 – 6.95 µm, truncate.
**G7**	Basidiocarp sessile. Pileus surface medium-brown, matte, 13.5 cm in length, 10 cm wide, dimidiate. Pileus imbricate, rugose, with concentric undulations and fissures. Context medium-brown, spongy texture, and absent melanoid band. Tube layer dark-brown, pores slightly oval, 196.87 mm, 5 (4: 5) pores per mm, 75.9 mm between pores, and 242.23 mm between pore axes.
**G8**	Basidiocarp sessile, laterally attached. Pileus surface medium to dark-brown, with tons of yellow and red, laccate, 6 cm in length, 5 cm wide, and 2 cm thick at the base, reniform. Pileus imbricate, rugose, with concentric undulations and fissures. Regular cream margin. Context dark brown, spongy texture near the bottom and woody near the top, 1.6 cm thick, and absent melanoid band. Tube layer dark-brown, 0.3 cm thick with white strikes near the outer edge. Pore surface cream, pores round, 105.13 mm, 5 (5: 6) pores per mm, 47.7 mm between pores, and 195.35 mm between pore axes. Basidiospores obovate, 8.39 – 10.98 µm x 6.49 – 8.31 µm, truncate.
**G9**	Basidiocarp stipitate, laterally attached. Stipe projecting 7 cm in length and 2.8 thick, dark-brown to purple. Pileus surface medium to dark-brown, with tons of yellow and red, laccate, 9 cm in length, 5.5 cm wide, and 3 cm thick at the base, reniform. Pileus imbricate, smooth, with concentric undulations, with no fissures. Regular light-brown margin. Context medium-brown, spongy texture, 2 cm thick, and present melanoid band. Tube layer medium-brown, 0.4 cm thick. Pore surface cream, pores round, slightly oval, 181.13 mm, 6 (5: 6) pores per mm, 68.04 mm between

	pores, and 217.56 mm between pore axes. Basidiospores obovate, 7.1 – 12.12 µm x 5 – 9.19 µm, truncate.
**G10**	Basidiocarp stipitate, laterally-dorsal attached. Stipe projecting 2.5 cm in length and 1.5 thick, dark-brown to reddish. Pileus surface dark-brown reddish, becoming light-cream near edge, laccate, 9 cm in length, 6.5 cm wide, and 3 cm thick at the base, suborbicular. Pileus imbricate, smooth and with fissures. Regular light-brown margin. Context dark to light-brown, spongy texture, 3 cm thick, and absent melanoid band. Tube layer medium-brown, 0.1 cm thick. Pore surface cream, pores round, slightly oval, 125.2 mm, 4 (4: 5) pores per mm, 158.16 mm between pores, and 283.5 mm between pore axes. Basidiospores obovate, 8.33 – 11.98 µm x 4.69 – 6.39 µm, truncate.
**G11**	Basidiocarp stipitate, laterally attached. Stipe projecting 3 cm in length and 1.5 cm thick, dark-brown to reddish. Pileus surface dark-brown reddish, laccate, 3.4 cm in length, 2.7 cm wide, and 0.9 cm thick at the base, spatulate. Pileus imbricate, smooth, with concentric undulations, with no fissures. Regular cream margin. Context dark brown, spongy texture, 0.6 cm thick, and present melanoid band. Tube layer medium-brown, 0.2 cm thick. Pore surface cream, pores round, 114.27 mm, 6 (5: 6) pores per mm, 67.21 mm between pores, and 170.39 mm between pore axes. Basidiospores obovate, 8.77 – 11.46 µm x 5.81 – 8.37 µm, truncate.
**G12**	Basidiocarp stipitate, dorsally attached. Stipe projecting 2 cm in length and 1.8 cm thick, dark-brown to reddish. Pileus surface dark-brown, with tons of yellow and red, laccate, 6.2 cm in length, 4 cm wide, and 3.5 cm thick at base, obovate. Pileus ungulate, smooth, with concentric undulations, with no fissures. Regular cream margin. Context dark brown, spongy texture, 3.5 cm thick, and absent melanoid band. Tube layer dark-brown, 0.1 – 0.3 cm thick. Pore surface cream, pores round, slightly oval, 175.83 mm, 5 (5: 6) pores per mm, 54.76 mm between pores, and 195.73 mm between pore axes. Basidiospores obovate, 4.85 – 10.87 µm x 4.88 – 7.37 µm, truncate.
**G13**	Basidiocarp surface dark-brown to black, laccate, larger than 5.5 cm in length, and 1.4 cm thick at base Pileus imbricate, rugose, with concentric undulations, with no fissures, dark-brown to black margin (old specimen). Context dark-brown near bottom and medium-brown near pileus, woody texture, 0.4 cm thick, and present melanoid band. Tube layer dark-brown, 1 cm thick. Pore surface medium-brown, pores round, 109.67 mm, 6 pores per mm, 78.66 mm between pores, and 180.38 mm between pore axes. Basidiospores obovate, 8.67 – 11.18 µm x 6.07 – 6.69 µm, truncate.
**G14**	Basidiocarp sessile, laterally attached. Pileus surface medium-brown, matte, larger than 4.5 cm in length, 3 cm wide, and 1.4 cm thick at the edge, rugose, with concentric undulations dark-brown margin (old specimen). Context dark brown, spongy texture, 0.7 cm thick, and present melanoid band. Tube layer dark-brown, 0.7 cm thick with white strikes near the outer edge. Pore surface cream (dark-brown where scratched), pores round, 201.64 mm, 4 pores per mm, 60.94 mm between pores, and 246.4 mm between pore axes. Basidiospores ovate, 8.25 – 9.91 µm x 5.54 – 6.82 µm, truncate.
**G15**	Basidiocarp sessile, laterally attached. Pileus surface dark-brown reddish, laccate, 13 cm in length, 9 cm wide, and 2.5 cm thick at the base, reniform. Pileus imbricate, rugose, with concentric undulations, with no fissures. Irregular cream margin. Context dark brown, spongy texture, 1.1 cm thick, and present melanoid band. Tube layer dark-brown, 1.2 cm thick. Pore surface cream (dark-brown where scratched), pores round, 141.06 mm, 6 (5: 6) pores per mm, 57.3 mm between pores, and 180.81 mm between pore axes. Spores ovate to obovate, 8.54 – 11.02 µm x 6.18 – 6.85 µm, truncate.
**G16**	Basidiocarp stipitate, laterally attached, dark-brown to purple. Pileus surface dark-brown reddish, laccate, 7 cm in length, 5 cm wide, and 1 cm thick at the base, reniform. Pileus imbricate, smooth, with concentric undulations, with no fissures. Regular medium-brown margin. Context dark brown, spongy texture, 1 cm thick, and absent melanoid band. Tube layer dark-brown, 0.3 cm thick. Pore surface medium-brown 88.85 mm, 8 (7: 8) pores per mm, 57.66 mm between pores, and 150.5 mm between pore axes.
**G17**	Basidiocarp sessile, laterally-dorsal attached, dark-brown to reddish. Pileus surface dark-brown reddish, laccate, and 1.4 cm thick at the base, reniform. Pileus imbricate, smooth, with concentric undulations, with no fissures dark-brown margin. Context dark-brown near bottom and medium-brown near pileus, spongy texture, 0.6 cm thick, and present melanoid band. Tube layer dark-brown, 1.1 cm thick. The pore surface was dark-brown to black, round, 92.85 mm, 5 (4: 5) pores per mm, 126.66 mm between pores, and 213.91 mm between pore axis. Basidiospores 9.85 – 11.93 µm x 5.87 – 7.43 µm, truncate.
**G18**	Basidiocarp stipitate, laterally attached. Stipe projecting 15 cm in length and 3 cm thick, dark-brown to black. Pileus surface dark-brown to black, laccate, 10 cm in length, 8 cm wide, and 1 cm thick at the base, reniform. Pileus imbricate, rugose, with concentric undulations, with no fissures. Regular light-brown margin. Context dark brown, woody texture, 0.3 cm thick, and absent

	melanoid band. Tube layer dark-brown, 0.5 cm thick with clear white strikes near the outer edge. Pore surface cream, pores round, 156.56 mm, 5 (5: 6) pores per mm, 55.22 mm between pores, and 205.44 mm between pore axes. Basidiospores 8.99 – 11.53 µm x 5.19 – 6.9 µm, truncate.
**G19**	Basidiocarp surface dark-brown reddish, laccate, and 2 cm thick in the middle. Rugose, with concentric undulations. Irregular dark-brown, reddish margin. Context dark-brown near bottom and medium-brown near pileus, spongy texture, 0.7 cm thick, and present melanoid band. Tube layer dark-brown, 1 cm thick, with white strikes near the outer edge. Pore surface cream, pores round, slightly oval, 182.93 mm, 4 (4: 5) pores per mm, 69.4 mm between pores, and 216.78 mm between pore axes. Basidiospores 9.62 – 11.74 µm x 5.76 – 8.46 µm, truncate.
**G20**	Basidiocarp stipitate, laterally attached. Stipe projecting 2.5 cm in length and 1 cm thick, dark-brown to reddish. Pileus dark-brown with tons of red and yellow, laccate, 2.4 cm in length, 2.5 cm wide, and 1 cm thick at base, spatulate. Pileus imbricate, smooth, with concentric undulations, with no fissures. Regular light-brown margin. Context medium-brown near bottom and light-brown near pileus, spongy texture, 0.9 cm thick, and present melanoid band. Tube layer dark-brown, 0.1 cm thick. Pore surface cream, pores round, 181.82 mm, 5 (4: 5) pores per mm, 38.58 mm between pores, and 194.08 mm between pore axes. Basidiospores 8.35 – 11.7 µm x 5.16 – 6.93 µm, truncate.
**G21**	Basidiocarp surface dark-brown yellowish, matte, larger than 3.5 cm in length, 4 cm wide, and 3 cm thick at middle, rugose. Context dark-brown, with tons of yellow, woody texture, 2 cm thick, and absent melanoid band. Tube layer medium-brown yellowish, 0.6 cm thick with yellow strikes near the outer edge. Pore surface dark-brown yellowish, round, 8 (7: 9) pores per mm. Basidiospores 7.03 – 12.31 µm x 4.44 – 8.94 µm, truncate.
**G22**	Basidiocarp laterally attached. Pileus dark-brown yellowish, laccate, 3.5 cm in length, 2 cm wide, and 1.5 cm thick at the base, spatulate. Pileus imbricate, smooth, with concentric undulations. Regular light-brown margin. Context light-brown, spongy texture, 1.5 cm thick, and absent melanoid band. The young specimen did not develop a tube layer.
